# Reference gene selection for qRT-PCR analysis of flower development in *Lagerstroemia indica* and *L*. *speciosa*

**DOI:** 10.1371/journal.pone.0195004

**Published:** 2018-03-26

**Authors:** Tangchun Zheng, Zhilin Chen, Yiqian Ju, Han Zhang, Ming Cai, Huitang Pan, Qixiang Zhang

**Affiliations:** 1 Beijing Key Laboratory of Ornamental Plants Germplasm Innovation & Molecular Breeding, Beijing Forestry University, Beijing, China; 2 National Engineering Research Center for Floriculture, Beijing Forestry University, Beijing, China; 3 Beijing Laboratory of Urban and Rural Ecological Environment, Beijing Forestry University, Beijing, China; 4 Key Laboratory of Genetics and Breeding in Forest Trees and Ornamental Plants of Ministry of Education, Beijing Forestry University, Beijing, China; 5 School of Landscape Architecture, Beijing Forestry University, Beijing, China; Michigan State University, UNITED STATES

## Abstract

Quantitative real-time polymerase chain reaction (qRT-PCR) is a prevalent method for gene expression analysis, depending on the stability of the reference genes for data normalization. *Lagerstroemia indica* and *L*. *speciosa* are popular ornamental plants which are famous for the long flowering period. However, no systematic studies on reference genes in *Lagerstroemia* have yet been conducted. In the present study, we selected nine candidate reference genes (*GAPDH*, *TUA*, *TUB*, *18S*, *RPII*, *EF-1α*, *ATC*, *EIF5A* and *CYP*) and evaluated their expression stability in different tissues during floral development of *L*. *indica* and *L*. *speciosa* using four algorithms (geNorm, NormFinder, BestKeeper and, RefFinder). Results showed that *RPII* and *EF-1α* were the most stably expressed and suitable reference genes for both of *Lagerstroemia* species. Moreover, *ACT* exhibited high expression stability in *L*. *indica* and *GAPDH* was a suitable reference gene for *L*. *speciosa* in different flower development stages. *TUB* was an unsuitable reference gene for gene expression normalization due to significant variations in expression across all samples. Finally, we verified the reliability of the selected candidate reference genes by amplifying an *AGAMOUS* homolog (*LsAG1*) of *Arabidopsis thaliana*. This study provides a list of suitable reference genes, thereby broadening the genetic basis of the gene expression patterns in *Lagerstroemia* species.

## Introduction

qRT-PCR is a popular method to study gene expression with high sensitivity, specificity and adaptability to high throughput analyses [1–3]. It bases on the use of reference gene as the normalization of gene transcription levels requires the stability of the selected reference genes [4,5]. Traditional reference genes, including *18S rRNA* (*18S ribosomal RNA*), *GAPDH* (*glyceraldehyde-3-phosphate dehydrogenase*), *EF-1α* (*elongation factor-1-alpha*), *TUA* (*alpha-tubulin*), *TUB* (*beta-tubulin*), *UBQ* (*ubiquitin*), and *ACT* (*actin*), are widely used in plants [6]. Some new reference genes have shown high stability under diverse conditions, such as *PP2A* (*protein phosphatase 2A*), *CYP* (*cyclophilin*) and *EIF5A* (*eukaryotic translation initiation factor 5A*) [7]. However, the present reference genes (Such as *GAPDH*, *TUB*, *UBQ*, and *CYP*) may show variation in expression levels across species, tissues or treatments [8]. Therefore, identification of new reference genes is needed to cover the wide range of expression levels. Presently, there are no constantly expressed reference genes that can cover different development stages or the entire lifecycle of plants [8]. Therefore, it is imperative to identify species-specific, stage-specific and organ-specific reference genes for qRT-PCR.

*Lagerstroemia* belongs to the Lythraceae family, which contains more than 80 known species in southeastern Asia [9]. As woody ornamentals, *Lagerstroemia* species are famous for their long-lasting summer bloom, rich colors and abundant flower types. They are also favored with diverse habitats, various landscape applications and few serious pest or disease issues, making them ideal for breeding [10]. *L*. *indica* and *L*. *speciosa* are the spectacular species being widely used in the gardening and plant breeding programs. They are considered an ideal representative to study flower development in *Lagerstroemia* species.

Exploring the flower development in the higher plants has been an attractive research topic since antiquity [11]. The mechanism of flower development has been revealed by analyzing the expression patterns of the key genes involved in floral meristem identity [12]. However, in the case of *Lagerstroemia* species, the focus has mainly been on the investigations into the physiological and metabolic engineering characteristics [13–15]. Only a few studies have documented the transcription and expression of flower-related genes in *Lagerstroemia*. Primarily, *ACT* was used as a reference gene for expression analyses between a leaf-color-mutant and wild-type *L*. *indica* [16]. Moreover, two housekeeping genes, *cab-h1* and *cab-h8*, were used for expression analyses between two cultivated *Lagerstroemia* species [17]. Therefore, owing to no systematic analysis of suitable reference genes for *Lagerstroemia*, it is necessary to evaluate the stability of candidate reference genes in different growth stages and tissues in *Lagerstroemia*.

In the present study, nine candidate reference genes were selected for qRT-PCR analyses in *Lagerstroemia*, including *GAPDH*, *TUA*, *TUB*, *18S*, *RPII*, *EF-1α*, *ATC*, *EIF5A* and *CYP*, which had been shown to be stable in *Vernicia fordii* [18], *Prunus persica* [19], *Citrus* [20] and *Paeonia suffruticosa* [7]. The change in transcription level of these candidate genes was evaluated by qRT-PCR in four sets of samples: A) flower development stages of *L*. *indic*a, B) floral organs of *L*. *indica*, C) flower development stages of *L*. *speciosa*, D) floral organs of *L*. *speciosa*. The floral homeotic gene *AGAMOUS* (*AG*), a class C gene of the MADS-box transcription factor family is necessary for specification and development of stamen and carpels along with floral meristem determinacy. In order to verify the authenticity and accuracy of the selected reference genes, the tissue-specific expression of *LsAG1*, an *AGAMOUS* homolog (*AtAG1*, AT4G18960) of *Arabidopsis thaliana*, was analyzed in all sample groups.

## Materials and methods

### Plant materials

**Fig 1:**
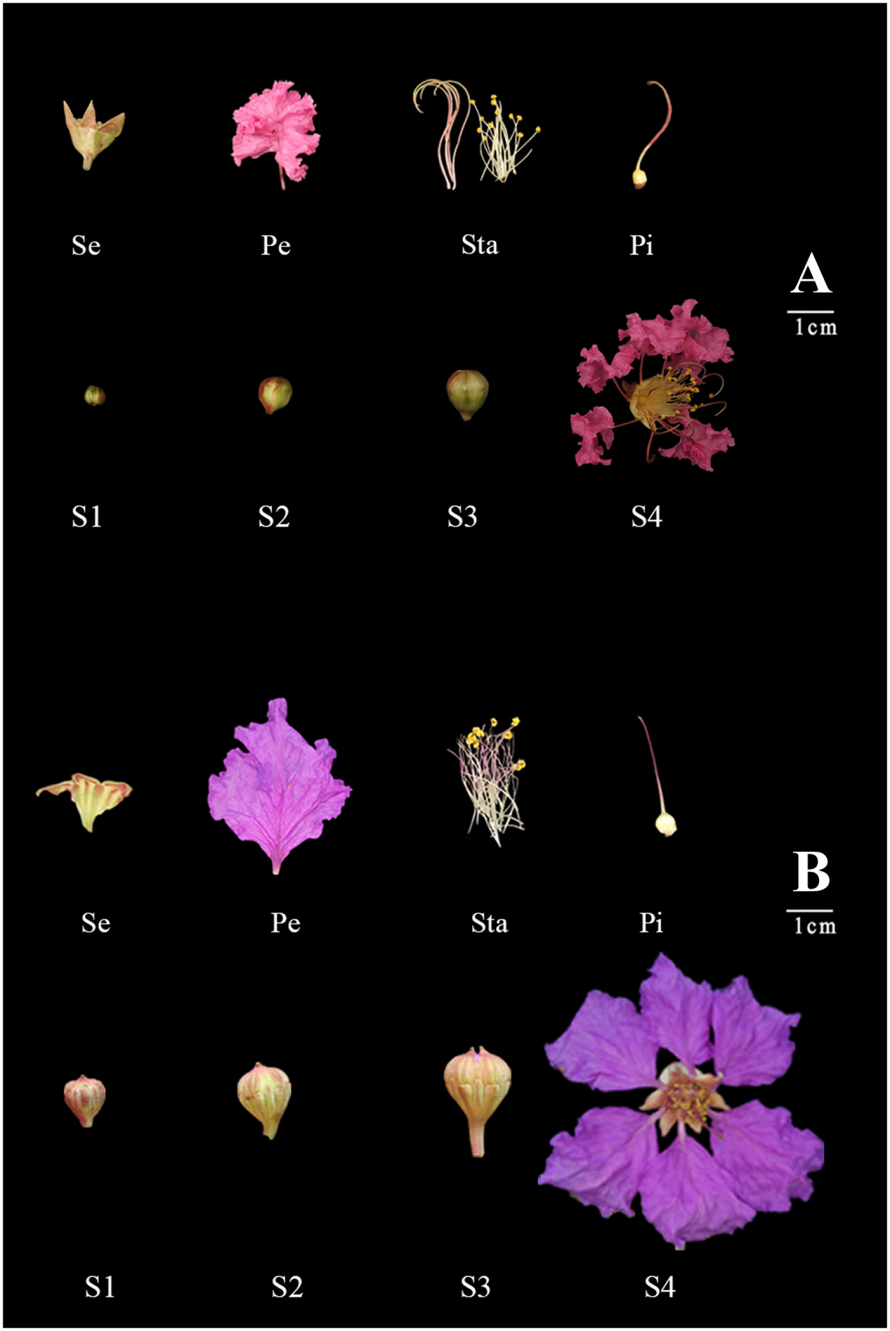
Flower samples and developmental stage of *L*. *indica* and *L*. *speciosa*. **A** = *L*. *indica*; **B** = *L*. *speciosa*; Se = Sepal; Pe = Petal; Sta = Stamen; Pi = Pistil; S1 = Stage 1; S2 = Stage 2; S3 = Stage3; S4 = Stage 4.

Flower samples of *L*. *indica* and *L*. *speciosa* were taken from the campus of Beijing Forestry University and the nursery garden of Guangxi Academy of Forestry (Nanning, Guangxi, China), respectively. The flower samples were collected at different developmental stages (Fig 1): tight bud (S1), loose bud (S2), bud with cleft (S3), and fully opened flower with exposed anthers (S4). Sepals (Se), petals (Pe), stamens (Sta) and pistils (Pi) were excised from flowers at full opening stage (S4). Samples were collected in triplicates with each repeat comprising samples from at least 10 flowers, and three biological replicates were performed for each tissue and developmental stage. Plant tissues were immediately frozen in liquid nitrogen and stored at -80°C for further analysis.

### RNA extraction and cDNA synthesis

Total RNA was isolated from all samples using MiniBEST Plant RNA Extraction Kit (TaKaRa, Dalian, China). Concentration and purity of total RNA were assessed by a micro-spectrophotometer (NanoDrop 2100C, Thermo, Wilmington, DE, USA). The absorbance ratio of samples was greater than 1.8 both at *OD*_260_/_280_ and *OD*_260_/_230_. 28S/18S ratio between 1.8 and 2.0 was used for subsequent experiments. 1 μg aliquot of total RNA, treated with DNase I (Invitrogen, Carlsbad, CA, USA), was reverse-transcribed using PrimeScript RT reagent Kit (TaKaRa, Dalian, China) according to operating manual. All cDNAs were diluted 1:10 with ddH_2_O for further analyses.

### Identification of candidate reference genes and primer designing

In our previous study, six cDNA libraries (two individual shoot tips samples, three biological repeats, respectively) were constructed and sequenced by using the Illumina RNA-Seq method (SRA accession: SRP132114). A total of 45,929 unigenes were annotated in NCBI non-redundant protein database, and further used for mining candidate reference genes based on expression stability. To estimate expression stability of each gene, the values of mean value (MV), standard Deviation (SD), and coefficient of variation (CV), were calculated for each gene based on fragments per kilobase of transcript per million fragments mapped (FPKM). The genes that had both a mean of FPKM above 25 and a CV below 0.1 were considered to be stably expressed. Candidate reference genes were selected from the homologous of traditional housekeeping genes previous used for flower development according to gene NR annotation. A set of nine candidate reference genes (S1 File) was selected based on transcriptome data to evaluate the most suitable candidate reference genes for qRT-PCR among different plants. All candidate reference genes were cloned into pMD18-T vector (TaKaRa), the positive colonies were selected and the recombinants were identified by Sangon Biotech (Shanghai) Co. Ltd. (Shanghai, China) for sequencing. All the primers for qRT-PCR were designed using Primer5 software. Each primer pair was performed by experimental evaluation and was accepted if all following conditions were true: (1) product PCR reaction using cDNA as a template was pecific, (2) reaction using genomic DNA as a template gave no product, and (3) the efficiency of a real time PCR reaction was between 90–110% (Table 1).

**Table 1:** Genes and primer sets used for qRT-PCR in *L*. *indica* and *L*. *speciosa*.

Gene symbol	Gene description	Genebank ID	Primer sequences (forward primer/reverse primer, 5'-3')	Product length(bp)	Melting temperature (°C)	Amplification efficiency (%)	Correlation coefficient R^2^
***GAPDH***	*Glyceraldehyde-3-phosphate*	MG704143	AGGATTGGAGAGGTGGTAGGGC/CAACAGTGGGGACACGGAAAG	136	60	104.01%	0.996
***TUA***	*Alpha-tubulin*	MG704144	CTCGTGCTGTTTTTGTTGACCT/TCTCTTTCCCAATTGTGTAGTG	153	60	103.18%	0.986
***TUB***	*Beta-tubulin*	MG704145	TCCAGAACAAGAACTCCTCCTA/GCTGTAAACTGCTCGCTCACCC	163	60	99.86%	0.977
***18S***	*18S ribosomal RNA*	MG704137	GACTCAACACGGGGAAACTTACC/CAGACAAATCGCTCCACCAAC	123	60	90.50%	0.996
***EIF****5A*	*Eukaryotic translation initiation factor 5A*	MG704142	GGGACGGTTTTTGATGACGA/CGGACGAGGAGCACCACTTC	113	60	90.82%	0.984
***EF-1α***	*Elongation factor-1α*	MG704141	GACTGTGCTGTGCTCATC/GTGGCATCCATCTTGTTG	146	60	102.68%	0.977
***ATC***	*Actin*	MG704138	ACCGGTGTTATGGTTGGTATG/CCGTGCTCAATGGGATACTT	101	60	99.81%	0.988
***CYP***	*Cyclophilin*	MG704140	GTTCGCTGACGAGAACTTCA/CTTAGCGGTGCAGATGAAGAA	109	60	100.54%	0.981
***RPII***	*RNA polymerase II*	MG704139	GCGGGTCCTCGATGTTCTAG/GTCCGAGAGATTCAGCCGAG	130	60	109.71%	0.977
***LsAG1***	*AGAMOUS* homologue	MG704146	GTGGAGCTGAAGAGGATAGA/GAGAAGATGATGAGAGCAACC	131	60	106.99%	0.993

### Real-time qRT-PCR assays

The cDNA template (2 μL, equivalent to 10 ng total RNA) was used in the qRT-PCR with SYBR Premix Ex*Taq* II (TaKaRa) and qTower2.0 Real-time PCR System (Analytik Jena AG, Jena, Germany) according to the laboratory manual. The 20-μL reaction volume contained 2 μL of diluted cDNA, 0.5 μL of forward and reverse primers (25 μM), 10 μL of 2×SYBR Premix *ExTaq* and 7 μL of ddH_2_O. The amplification was conducted under the following conditions: 95°C for 30 s, 40 cycles of 95°C for 5 s and 60°C for 30 s, heating from 60°C to 95°C with a 0.5°C w/s increment to determine melting curves. Each reaction was done in triplicate to ensure reproducibility of results. Expression levels were calculated from the cycle threshold according to the delta-delta CT method.

The primer amplification efficiency was determined from a standard curve generated by serial dilutions of cDNA (10-fold each) for each gene in triplicate. Correlation coefficients (R^2^ values) and amplification efficiencies (E) for all primer pairs were calculated from the slope of the regression line by plotting mean *Cq* values against the log cDNA dilution factor in Microsoft Excel using the equation E (%) = (10^[-1/slope]^ -1)×100.

### Statistical analysis

Four different applications were used to calculate and rank the stability of the nine candidate genes, including: geNorm [21], NormFinder [22], BestKeeper [23], and RefFinder (http://150.216.56.64/referencegene.php). As a visual Excel tool, geNorm calculates gene expression stability (*M*) based on the average pairwise variation (*V*). The default value of *M* is 1.5; stably expressed genes have a value below 1.5 [21]. NormFinder is a program which calculates intra- and intergroup variations and combines the two results into a stability value of each candidate gene, the reference genes with lower average expression values are more stable. BestKeeper uses the coefficient of variance (*CV*) and the standard deviation (*SD*) of the *Cq* values to rank the reference genes. The stability of a reference gene is indicated by low *CV* and *SD* values [*CV*±*SD*]. If the *SD* value exceeds 1.0, the reference gene should be excluded from gene expression normalization. RefFinder is an online tool used to verify the accuracy of the calculation. The program includes geNorm, NormFinder, BestKeeper and delta CT to analyze the stability of reference genes comprehensively.

### Assessment of normalization

*AGAMOUS* is floral homeotic gene that encodes a MADS domain transcription factor and regulates floral meristem and carpel and stamen identities in plants [6,24,25]. To verify the authenticity and accuracy of the nine candidate reference genes, we evaluated the expression pattern of *LsAG1* gene (An *AGAMOUS* homolog in *L*. *speciosa*). Primers for *LsAG1* are presented in Table 1.

## Results

### Primer specificity and amplification efficiency analysis

We first determined the specificity and amplification efficiency of the primers by gel electrophoresis and melting curve analyses. All primers amplified a single band of expected size (S1 Fig). The presence of a single peak for each gene indicates the specificity of primers (S2 Fig), while no-template controls showed no peaks. The qRT-PCR amplification efficiency of the nine candidate reference genes ranged between 90.50% (*18S*) and 109.71% (*RPII*); correlation coefficients varied from 0.977 (*TUB*/*RPII*) to 0.996 (*GAPDH*) (Table 1). Therefore, all the primers were available for further experiments.

### Expression profiling of candidate reference genes

**Fig 2:**
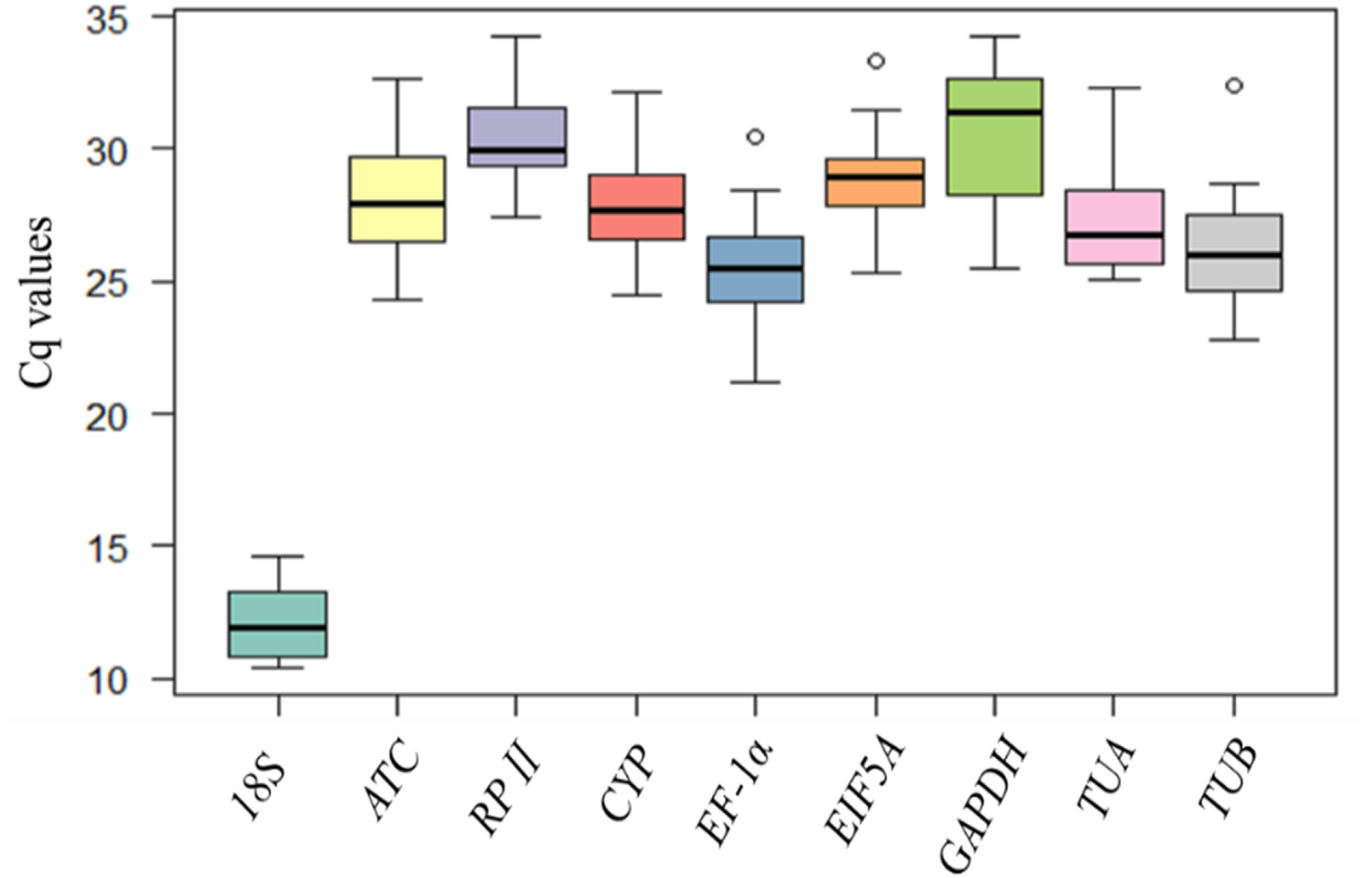
Expression levels of candidate reference genes across all samples of *L*. *indica* and *L*. *speciosa* at different flower development stages. The lines across the box are the medians, the boxes depicts the 25/75 percentiles, the whiskers represent the 95% confidence intervals, and the dots are outliers.

qRT-PCR for transcript profiling was performed with primers of the nine candidate reference genes in the 16 sample sets of *L*. *indica* and *L*. *speciosa*. *Cq* value presented the expression level of nine reference genes (Fig 2). The average *Cq* values varied from 12.03 to 30.47 across all samples, which indicated different expression levels. *18S* had the lowest *Cq* (12.03), showing the highest level of expression. *GAPDH* had the highest *Cq* (30.47), which had the lowest level of expression. None of these candidate reference genes had a completely constant level of expression across all samples. In conclusion, simple comparison of the raw *Cq* values could not provide enough information regarding expression stability. Therefore, we used four different statistical programs to evaluate the stable reference genes for *Lagerstroemia*.

### Expression stability of the candidate reference genes

To ensure accuracy, we analyzed all samples in four sets. Set A and C consisted of four development stages of *L*. *indica* and *L*. *speciosa*, respectively. Set B and D comprised of four floral organs from *L*. *indica* and *L*. *speciosa*, respectively. geNorm, NormFinder, BestKeeper and RefFinder were used to assess the expression level of nine candidate reference genes.

#### geNorm analysis

**Fig 3:**
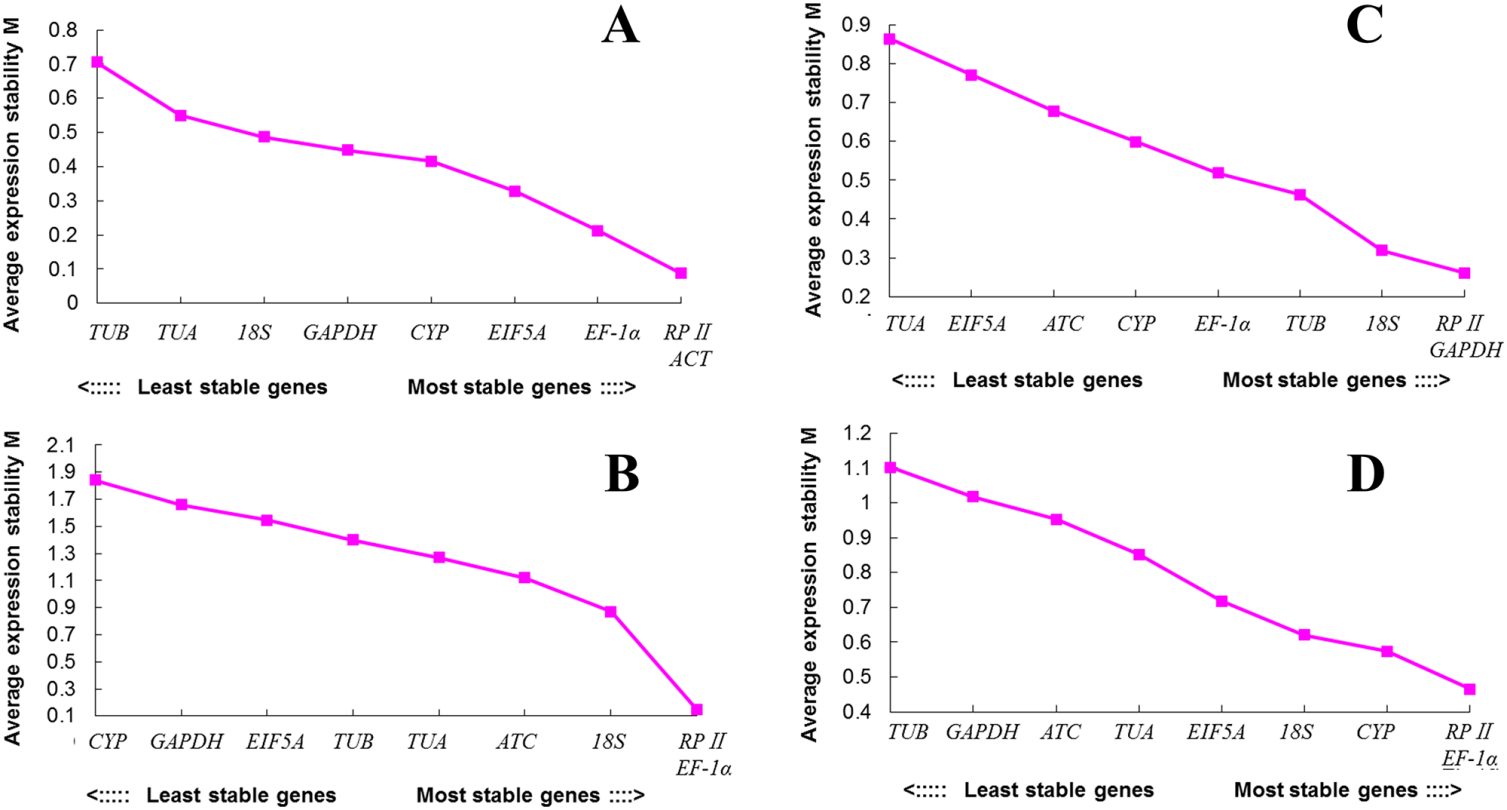
Average expression stability and ranking of nine candidate reference genes evaluated by geNorm software. The average expression stability value (*M*) was calculated following stepwise exclusion of the least stable gene across all samples. (**A**) Different flower development stages of *L*. *indica*, (**B**) Different flower organs of *L*. *indica*, (**C**) Different flower development stages of *L*. *speciosa*, (**D**) Different flower organs of *L*. *speciosa*. The most stable genes are on the right, while the least stable ones are on the left.

Average expression stability (*M*) of reference genes was calculated and ranked by GeNorm program. The ranking order is depicted in Fig 3. Most genes were stable with *M*-value below 1.5, except for *CYP* (*M* = 1.84), *GAPDH* (*M* = 1.66) and *EIF5A* (*M* = 1.55) in set B. In set A, *RPII* and *ACT* were the most stable genes with an *M*-value of 0.08, while *TUB* had a high value (0.70). In set B, *RPII* and *EF-1α* were the most stable genes (*M* = 0.15), while *CYP* was the least one with an *M*-value of 1.81. In set C, *RPII* and *GAPDH* performed well with an *M*-value of 0.26 and *TUA* exhibited a high *M*-value (0.86). In set D, *M*-value of *RPII* and *EF-1α* was 0.47, showing the highest stability, while *TUB* was the least stable gene with an *M*-value of 1.10. In conclusion, *RPII* and *EF-1α* ranked best across all samples with the highest stability.

**Fig 4:**
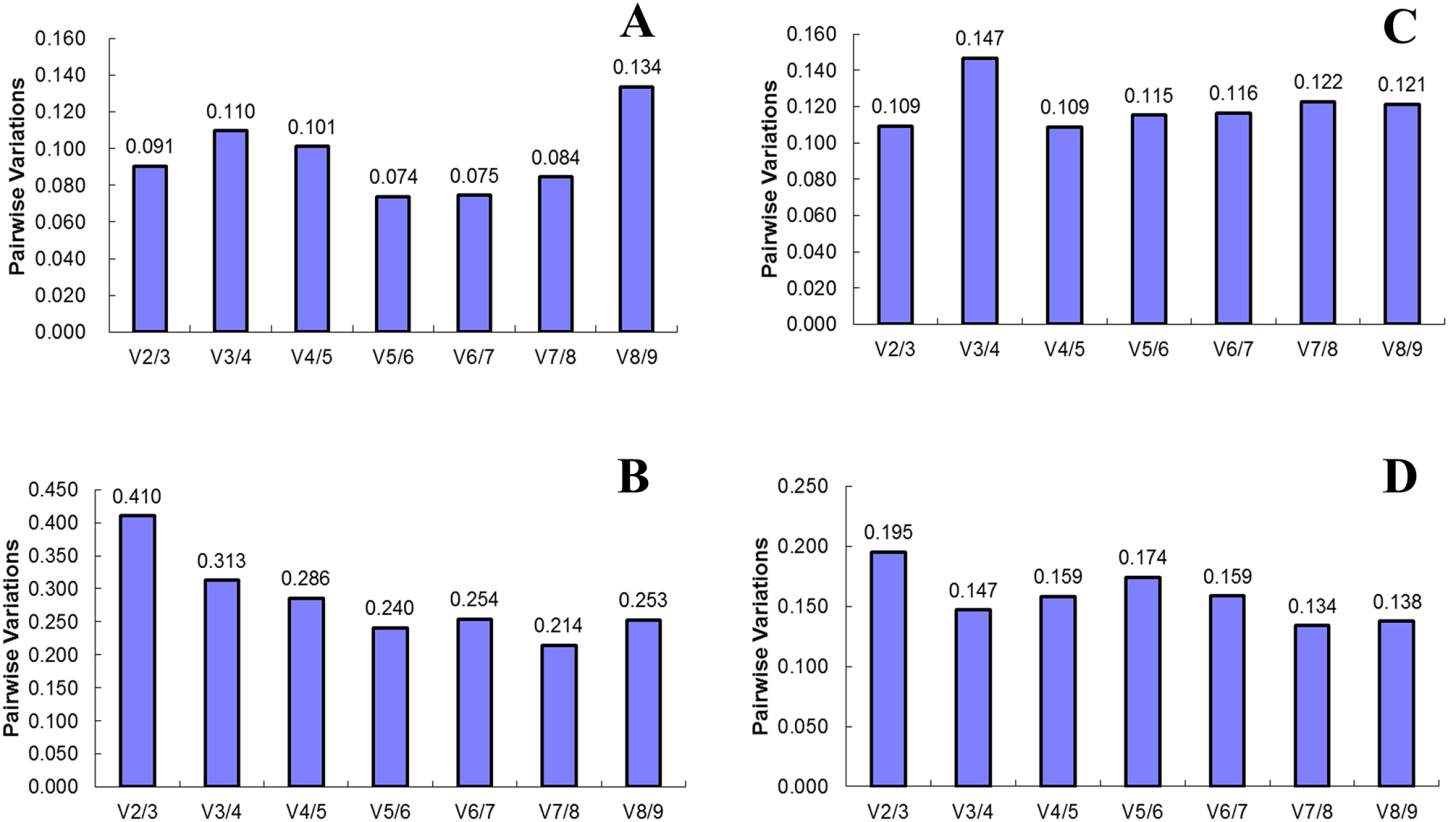
Pairwise variation calculated by geNorm between V_n_ and V_n + 1_ to determine the minimum number of reference genes required for accurate normalization in four different groups. (**A**) Different flower development stages of *L*. *indica*, (**B**) Different flower organs of *L*. *indica*, (**C**) Different flower development stages of *L*. *speciosa*, (**D**) Different flower organs of *L*. *speciosa*. The cut off value is 0.150, below which the inclusion of an additional reference gene is not required.

To determine the optimal number of reference genes in each experimental condition, pairwise variation (*V*) was calculated using geNorm by applying a cutoff value of 0.150. A pairwise variation analysis showed that the optimal number of reference genes may be different for distinct samples. If V_n_/V_n+1_ <0.15, the number of the most suitable internal reference genes is n, while if V_n_/V_n+1_ is > 0.15, the number of the most suitable internal reference genes is n + 1. As shown in Fig 4, *V2/V3* was less than 0.15 both in set A and C (with values of 0.091 and 0.109, respectively), which indicated that only two reference genes is suffieient for normalizing gene expression. The pairwise variation in set D was *V2/3* 0.195, which is above the cutoff of 0.150, and the addition of next reference gene decrease this value to 0.147 (*V3/4*), implying that proper normalization required at least three reference genes.

#### NormFinder analysis

The stability of reference genes was also evaluated by NormFinder program based on the intra-and intergroup variations among all genes. As shown in Table 2, the ranking order determined by this program was consistent across the results generated by geNorm with little differences. For example, *EIF5A* was the most stable gene in set A when determined by NormFinder, whereas it was ranked fourth by geNorm. In set D, NormFinder determined *EF-1α* and *EIF5A* as the most stable genes, whereas *EIF5A* was ranked fifth by geNorm. Considering the results of all sets, the most stable reference genes were still *RPII* and *EF-1α*.

**Table 2:** Expression stability of the reference gene calculated by NormFinder for *L*. *indica* and *L*. *speciosa*.

Ranking	Set A		Set B		Set C		Set D	
	Gene Name	Stability Value	Gene Name	Stability Value	Gene Name	Stability Value	Gene Name	Stability Value
**1**	*EIF5A*	0.175	*RPII*	0.346	*RPII*	0.036	*EF-1α*	0.141
**2**	*ATC*	0.261	*EF-1α*	0.513	*EF-1α*	0.344	*EIF5A*	0.330
**3**	*RPII*	0.342	*18S*	1.029	*GAPDH*	0.348	*RPII*	0.539
**4**	*18S*	0.344	*ATC*	1.057	*TUB*	0.404	*ATC*	0.847
**5**	*CYP*	0.435	*GAPDH*	1.394	*18S*	0.447	*TUA*	0.853
**6**	*GAPDH*	0.506	*TUB*	1.595	*ATC*	0.706	*CYP*	0.877
**7**	*EF-1α*	0.540	*EIF5A*	1.639	*CYP*	0.762	*GAPDH*	0.972
**8**	*TUA*	0.594	*TUA*	1.673	*EIF5A*	1.033	*18S*	1.041
**9**	*TUB*	1.195	*CYP*	2.235	*TUA*	1.071	*TUB*	1.212

Set A = flower development stages of *L*. *indica*, set B = flower organs of *L*. *indica*, set C = flower development stages of *L*. *speciosa*, set D = flower organs of *L*. *speciosa*.

#### Bestkeeper analysis

Bestkeeper ranks according to the standard deviation (*SD*) and the coefficient of variation (*CV*). The ranking order was different from the results exhibited by geNorm and NormFinder. As shown in Table 3, the *SD*-values of *RPII* and *EF-1α* were mostly less than 1.0 in set A, C and D, indicating these two genes were suitable for gene expression normalization. However, the *SD*-values of most of the genes were higher than 1.0 in set B. But *RPII* was ranked third and *EF-1α* was fourth, making them reliable reference genes as compared to others.

**Table 3:** Expression stability of the reference gene calculated by Bestkeeper for *L*. *indica* and *L*. *speciosa*.

Ranking	Set A		Set B		Set C		Set D	
	Gene Name	*CV*±*SD*	Gene Name	*CV*±*SD*	Gene Name	*CV*±*SD*	Gene Name	*CV*±*SD*
**1**	*EF-1α*	2.75±0.65	*EIF5A*	1.60±0.48	*CYP*	1.73±0.50	*CYP*	1.51±0.41
**2**	*RPII*	2.51±0.78	*RPII*	4.97±1.55	*GAPDH*	2.68±0.87	*RPII*	1.77±0.52
**3**	*GAPDH*	2.96±0.80	*EF-1α*	5.87±1.60	*TUA*	3.22±0.86	*EF-1α*	2.91±0.72
**4**	*ATC*	3.02±0.80	*GAPDH*	6.09±1.88	*RPII*	3.48±1.06	*18S*	2.95±0.32
	*CYP*	3.10±0.87	*ATC*	6.67±1.96	*EF-1α*	3.55±0.96	*EIF5A*	3.68±1.02
**6**	*EIF5A*	3.92±1.07	*18S*	7.88±1.01	*TUB*	3.90±1.01	*TUA*	3.84±1.01
**7**	*TUA*	4.89±1.33	*TUA*	7.88±2.29	*EIF5A*	5.42±1.67	*GAPDH*	4.31±1.35
**8**	*18S*	9.86±1.16	*CYP*	8.28±2.30	*ATC*	5.51±1.63	*ATC*	5.52±1.51
**9**	*TUB*	7.01±1.84	*TUB*	9.19±2.51	*18S*	9.64±1.20	*TUB*	6.48±1.61

Set A = flower development stages of *L*. *indica*, set B = flower organs of *L*. *indica*, set C = flower development stages of *L*. *speciosa*, set D = flower organs of *L*. *speciosa*.

#### RefFinder analysis

Ref-Finder was used to combine the results drawn by the other three programs and to rank the nine candidate reference genes synthetically (Table 4). Though some differences exist among the results of the four programs, the most stable gene was basically identical. *RPII* ranked first in set A, B and C. *EF-1α* was the most stable gene in set D. *ACT* and *GAPDH* ranked second in set A and C, respectively. *TUB* was the least stable gene in set A and D.

**Table 4:** Expression stability of the reference gene calculated by Ref-finder for *L*. *indica* and *L*. *speciosa*.

Ranking		1	2	3	4	5	6	7	8	9
**Set A**	**G**	*RPII*	*ATC*	*EF-1α*	*EIF5A*	*CYP*	*GAPDH*	*18S*	*TUA*	*TUB*
	**N**	*EIF5A*	*ATC*	*RPII*	*18S*	*CYP*	*GAPDH*	*EF-1α*	*TUA*	*TUB*
	**B**	*EF-1α*	*RPII*	*GAPDH*	*ATC*	*CYP*	*EIF5A*	*TUA*	*18S*	*TUB*
	**R**	*RPII*	*ATC*	*EIF5A*	*EF-1-α*	*CYP*	*GAPDH*	*18S*	*TUA*	*TUB*
**Set B**	**G**	*RPII*	*EF-1α*	*18S*	*ATC*	*TUA*	*TUB*	*EIF5A*	*GAPDH*	*CYP*
	**N**	*RPII*	*EF-1α*	*18S*	*ATC*	*GAPDH*	*TUB*	*EIF5A*	*TUA*	*CYP*
	**B**	*EIF5A*	*RPII*	*EF-1α*	*GAPDH*	*ATC*	*18S*	*TUA*	*CYP*	*TUB*
	**R**	*RPII*	*EF-1α*	*18S*	*ATC*	*EIF5A*	*GAPDH*	*TUA*	*TUB*	*CYP*
**Set C**	**G**	*RPII*	*GAPDH*	*18S*	*TUB*	*EF-1α*	*CYP*	*ATC*	*EIF5A*	*TUA*
	**N**	*RPII*	*EF-1α*	*GAPDH*	*TUB*	*18S*	*ATC*	*CYP*	*EIF5A*	*TUA*
	**B**	*CYP*	*GAPDH*	*TUA*	*RPII*	*EF-1α*	*TUB*	*EIF5A*	*ATC*	*18S*
	**R**	*RPII*	*GAPDH*	*EF-1α*	*CYP*	*TUB*	*18S*	*TUA*	*ATC*	*EIF5A*
**Set D**	**G**	*RPII*	*EF-1α*	*CYP*	*18S*	*EIF5A*	*TUA*	*ATC*	*GAPDH*	*TUB*
	**N**	*EF-1α*	*EIF5A*	*RPII*	*ATC*	*TUA*	*CYP*	*GAPDH*	*18S*	*TUB*
	**B**	*CYP*	*RPII*	*EF-1α*	*18S*	*EIF5A*	*TUA*	*GAPDH*	*ATC*	*TUB*
	**R**	*EF-1α*	*RPII*	*EIF5A*	*CYP*	*18S*	*TUA*	*ATC*	*GAPDH*	*TUB*

G = geNorm; N = NormFinder; B = BestKeeper; R = Recommended comprehensive ranking. Set A = flower development stages of *L*. *indica*, set B = flower organs of *L*. *indica*, set C = flower development stages of *L*. *speciosa*, set D = flower organs of *L*. *speciosa*.

### Reference gene validation

*RPII* and *EF-1α* were suitable genes for normalization across all samples both in *L*. *indica* and *L*. *speciosa*. *ATC* ranked second and forth in set A and B, respectively, suggesting it a good reference gene for *L*. *indica*. However, it was unsuitable for *L*. *speciosa* because of poor performance in set C and D. *GAPDH* was unstable in set A, B and D, but it was ranked second in set C and might be considered a reference gene to study flower developmental stages of *L*. *speciosa*. *TUB* was the least stable gene in *Lagerstroemia* because of poor ranking across all samples.

**Fig 5:**
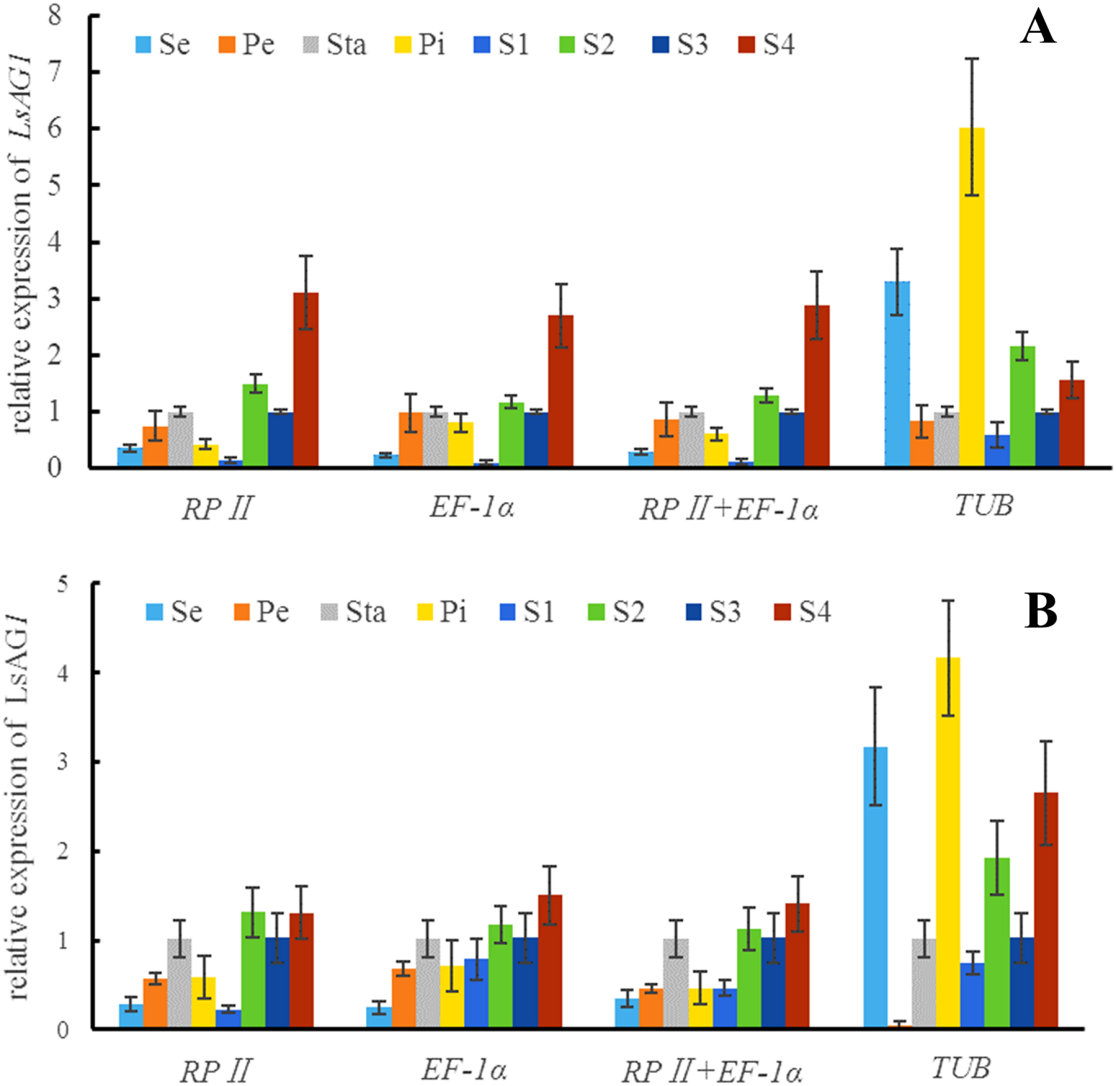
Relative expression level of *LsAG1*. (**A**) *L*. *indica*, (**B**) *L*. *speciosa*. Se = Sepal, Pe = Petal, Sta = Stamen, Pi = Pistil, S1 = Stage 1, S2 = Stage 2, S3 = Stage3, S4 = Stage 4.

In the case of normalization using the two most stable reference genes (*RPII* and *EF-1α*) separately and in combination (*RPII* + *EF-1α*) as an internal comparison, the expression of *LsAG1* gene showed similar trends with minor changes (Fig 5). The results showed that in *L*. *indica* and *L*. *speciosa*, the relative expression level of *LsAG1* was the highest in stamens and the lowest in sepals; *LsAG1* was up-regulated at S2 and then down-regulated at S3 and peaked at S4. When normalized with *TUB*, which was the least stable gene calculated by the four programs, the expression patterns were significantly different. Relative expression was abundant in sepals and pistils and peaked at S2.

## Discussion

The qRT-PCR method has become a popular and powerful tool to analyze the expression patterns of target genes, however, it requires proper reference genes for normalization [26]. qRT-PCR is convenient and fast to analyze the genetic regulation of plant growth and development, especially the floral development which is the key to diversification in plant kingdom. *Lagerstroemia* species are considered ideal to study the genetic regulation of key plant characteristics. *Lagerstroemia indica* and *L*. *speciosa* are popular ornamental plants and ideal breeding material to study floral development. Despite the immense importance, currently, there is no any universally-suitable reference gene for qRT-PCR analysis in the *Lagerstroemia* species. Several genes had been used as reference for *Lagerstroemia* in some prior studies focused on leaf color and disease resistance [16,17]. However, there is lack of candidate reference genes based on genotype, tissue type, developmental stage and experimental treatment [27]. Therefore, a systematic analysis of reference genes is required for research on flower development in *Lagerstroemia*. We identified nine candidate reference genes to test their stability in different tissues during flower development. Some of the candidate reference genes (*RPII*, *EF-1α*, and *Actin*) have previously been tested and are considered suitable candidate reference genes by researchers [7,16,18–20,28].

Based on previous findings, an accurate conclusion requires more than one program for analysis. Given that different statistical programs are based on distinct calculating principles, contradictory conclusion may be drawn from the same data. In the study of *Solanum melongena*, only two programs were used (geNorm and NormFinder) to evaluate the stability of gene expression, however, the results were inconsistent [29]. This suggests the use of at least three different algorithms to achieve reliable results [11]. Therefore, present study used four statistical programs (geNorm, NormFinder, BestKeeper and, RefFinder) to identify suitable reference genes for different floral organs and flower developmental stages in *Lagerstroemia*. The results of geNorm and NormFinder were similar but quite different from those exhibited by BestKeeper. In BestKeeper, *RPII* and *EF-1α* ranked fourth and fifth in set C, and *CYP* was the most stable gene in set D. But when we used RefFinder to summarize the results, *RPII* and *EF-1α* were still the most stable reference genes. Multi-algorithm analysis had been performed in other plants for the selection of reference genes under different situations, such as different color and flower developmental stages of *Paeonia suffruticosa* [7], different genotypes and abiotic-stress treatments of *Prunus mume* [6], different growth periods of *Vernicia fordii* [30] and hybrid detection of *Rosa* [31].

Based on findings of present research, it was necessary to select different housekeeping genes according to experimental design and sample material. *TUB* was recommended as a suitable reference gene in sunflower [32] and Chinese cabbage [33], however, it was confirmed to be the least stable gene in *Lagerstroemia* across all samples. Therefore, reference genes should be re-evaluated in different species. *RPII* and *EF-1α* were suitable genes for normalization both in *L*. *indica* and *L*. *speciosa*. These two genes have also been found in different tissues of *Prunus persica* [19], different genotypes and organs of *Citrus* [20], different tissues, organs and developmental stages of *Fraxinus* [34], *Capsicum annuum* [2], *Rhododendron micranthum* [35] and *Vernicia fordii* [18]. *ATC* was a good reference gene only in *L*. *indica* and *GAPDH* was appropriate for studying the individual flower developmental stages of *L*. *speciosa*. Thus, different reference genes are required based on the species and tissue types. This has also been shown in other plant species, such as *Actinidia chinensis* [36], *Pistacia vera* [37] and *Cicer* [38].

Using an unsuitable reference gene can affect expression pattern analyses and may lead to false results. To verify the selected reference genes, we assessed the relative expression level of *LsAG1* across all samples. When using *RPII*, *EF-1α* and their combination as reference genes, the expression patterns were similar. However, the results were quite different when the most unstable gene, *TUB*, was used for normalization, suggesting the importance of selecting suitable reference genes in experimental set-ups.

In summary, this research is the first systematic analysis of reference genes in *Lagerstroemia*. We used different tissues of flower developmental stages of *L*. *indica* and *L*. *speciosa*. Nine candidate reference genes (*GAPDH*, *TUA*, *TUB*, *18S*, *RPII*, *EF-1α*, *ATC*, *EIF5A*, and *CYP*) were screened and verified in qRT-PCR. Comprehensive assessment of expression stability by geNorm, NormFinder, BestKeeper, and RefFinder revealed that different genes should be used according to floral tissue types and developmental stages. In general, *RPII* and *EF-1α* were the most stable genes for *Lagerstroemia*. *ATC* was also a good reference gene for *L*. *indica* and *GAPDH* was suitable for studying the individual flower developmental stages of *L*. *speciosa*. *TUB* was the least stable gene and should not be applied as a reference gene in *Lagerstroemia*. The expression patterns of *LsAG1* further verified the importance of selection of suitable reference genes for normalization. Specific conclusions drawn from this research can give meaningful insights into the genetic basis of flower development in *Lagerstroemia*.

## Supporting information

S1 Fig Polymerase chain reaction amplification specificity of nine reference genes and *LsAG1* gene on a 1.0% agarose gel. (PDF)

S2 Fig Melting curves of nine candidate reference genes and *LsAG1* gene. (PDF)

S1 File All gene sequences used in paper. (DOCX)
