# Supplementary material for: Reference gene selection for qRT-PCR analysis of flower development in Lagerstroemia indica and L. speciosa
Source: PLoS One. 2018 Mar 26;13(3):e0195004. doi: 10.1371/journal.pone.0195004 (PMC5868847; doi:10.1371/journal.pone.0195004)
Supplement: S1 Fig — (PDF) [file pone.0195004.s001.pdf]

S1 Fig. Polymerase chain reaction amplification specificity of nine reference genes and *LsAG1* gene on a 1.0% agarose gel.

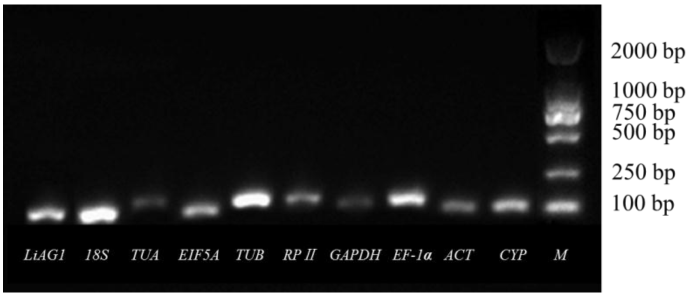

M=Maker DL 2000; *LsAG1* = An *AGAMOUS* homolog gene in *L. speciosa*; *18S* = *18S ribosomal RNA* gene; *TUA* = *Alpha tubulin gene*; *EIF5A* = *Eukaryotic translation initiation factor 5A* gene; *TUB* = Beta-tubulin gene; *RPII* = *RNA polymerase II* gene; *GAPDH* = *Glyceraldehyde-3-phosphate* gene; *EF-1α* = *Elongation factor 1-alpha* gene; *ACT* = *Actin* gene; *CYP* = *Cyclophilin* gene.
