# Supplementary material for: Reference gene selection for qRT-PCR analysis of flower development in Lagerstroemia indica and L. speciosa
Source: PLoS One. 2018 Mar 26;13(3):e0195004. doi: 10.1371/journal.pone.0195004 (PMC5868847; doi:10.1371/journal.pone.0195004)
Supplement: S2 Fig — (PDF) [file pone.0195004.s002.pdf]

S2 Fig. Melting curves of nine candidate reference genes and *LsAG1* gene.

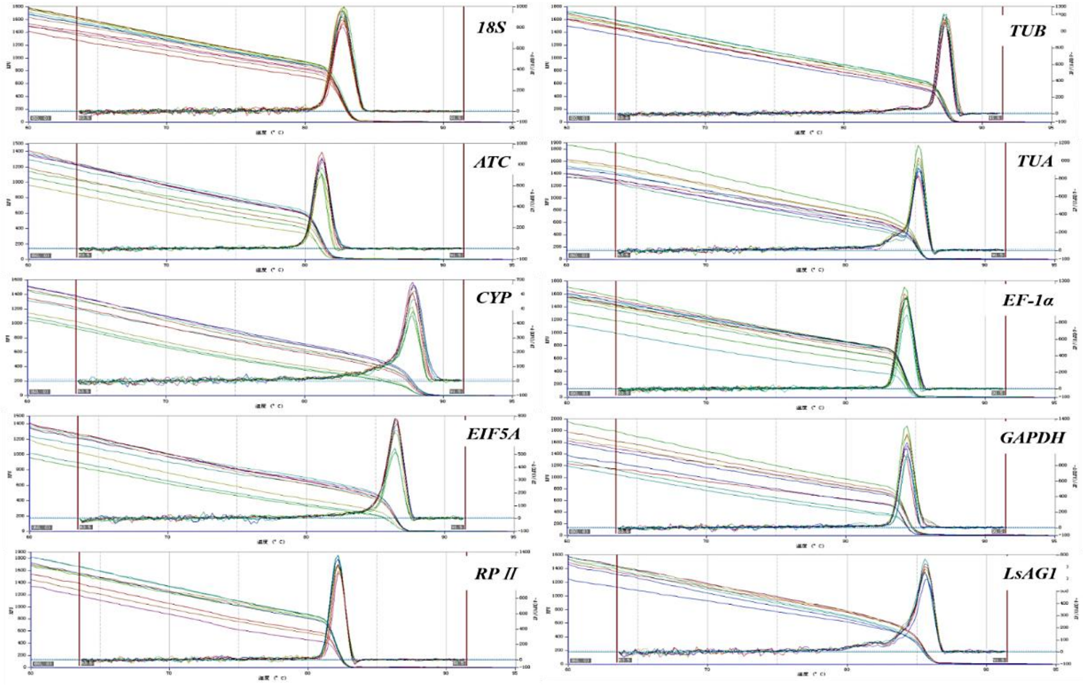

Temperature (℃) = the amplification temperature; RFU = the rate of change of the relative fluorescence units. *18S* = *18S ribosomal RNA* gene; *ACT* = *Actin* gene; *CYP* = *Cyclophilin* gene; *EIF5A* = *Eukaryotic translation initiation factor 5A* gene; *RPII* = *RNA polymerase II* gene; *TUB* = *Beta-tubulin* gene; *TUA* = *Alpha tubulin* gene; *EF-1α* = *Elongation factor 1-alpha* gene; *GAPDH* = *Glyceraldehyde-3-phosphate* gene; *LsAG1* = An *AGAMOUS* homolog gene *in L. speciosa*.
