# Supplementary material for: Reference gene selection for qRT-PCR analysis of flower development in Lagerstroemia indica and L. speciosa
Source: PLoS One. 2018 Mar 26;13(3):e0195004. doi: 10.1371/journal.pone.0195004 (PMC5868847; doi:10.1371/journal.pone.0195004)
Supplement: S1 File — (DOCX) [file pone.0195004.s003.docx]

> [Lagerstroemia indica] 18S ribosomal RNA

ATGCTTGTCTCAAAGATTAAGCCATGCATGTGCAAGTATGAACTAATTCAGACTGTGAAACTGCGAATGGCTCATTAAATCAGTTATAGTTTGTTTGATGGTATCTGCTACTCGGATAACCGTAGTAATTCTAGAGCTAATACGTGCAACGAACCCCGACTTCTGGAAGGGATGCATTTATTAGATAAAAGGTCGACGCGGGCTTTGCCCGATGCTCTGATGATTCATGATAACTTGACGGATCGCACGGCCATCGTGCCGGCGACGCATCATTCAAATTTCTGCCCTATCAACTTTCGATGGTAGGATAGTGGCCTACCATGGTGGTGACGGGTGACGGAGAATTAGGGTTCGATTCCGGAGAGGGAGCCTGAGAAACGGCTACCACATCCAAGGAAGGCAGCAGGCGCGCAAATTACCCAATCCTGACACGGGGAGGTAGTGACAATAAATAACAATACCGGGCTCATTGAGTCTGGTAATTGGAATGAGTACAATCTAAATCCCTTAACGAGGATCCATTGGAGGGCAAGTCTGGTGCCAGCAGCCGCGGTAATTCCAGCTCCAATAGCGTATATTTAAGTTGTTGCAGTTAAAAAGCTCGTAGTTGGACCTTGGGTTGGGTCGACCGGTCCGCCTTCAGGTGTGCACCGGTCGGCTCGTCCCTTCTACCGGCGATGCGCTCCTGGCCTTAATTGGCCGGGTCGTTCCTCCGGTGCTGTTACTTTGAAGAAATTAGAGTGCTCAAAGCAAGCCTACGCTCTGAATACATTAGCATGGGATAACATCATAGGATTCCGATCCTATTGTGTTGGCCTTCGGGATCGGAGTAATGATTAACAGGGACAGTCGGGGGCATTCGTATTTCATAGTCAGAGGTGAAATTCTTGGATTTATGAAAGACGAACAACTGCGAAAGCATTTGCCAAGGATGTTTTCATTAATCAAGAACGAAAGTTGGGGGCTCGAAGACGATCAGATACCGTCCTAGTCTCAACCATAAACGATGCCGACCAGGGATCAGCGGATGTTGCTTTTAGGACTCCGCTGGCACCTTATGAGAAATCAAAGTTTTTGGGTTCCGGGGGGAGTATGGTCGCAAGGCTGAAACTTAAAGGAATTGACGGAAGGGCACCACCAGGAGTGGAGCCTGCGGCTTAATTTGACTCAACACGGGGAAACTTACCAGGTCCAGACATAGTAAGGATTGACAGACTGAGAGCTCTTTCTTGATTCTATGGGTGGTGGTGCATGGCCGTTCTTAGTTGGTGGAGCGATTTGTCTGGTTAATTCCGTTAACGAACGAGACCTCAGCCTGCTAACTAGCTATGTGGAGGTACACCTCCACGGCCAGCTTCTTAGAGGGACTATGGCCGCTTAGGCCAAGGAAGTTTGAGGCAATAACAGGTCTGTGATGCCCTTAGATGTTCTGGGCCGCACGCGCGCTACACTGATGTATTCAACGAGTCTATAGCCTTGGCCGACAGGCCCGGGTAATCTTTGAAATTTCATCGTGATGGGGATAGATCATTGCAATTGTTGGTCTTCAACGAGGAATTCCTAGTAAGCGCGAGTCATCAGCTCGCGTTGACTACGTCCCTGCCCTTTGTACACACCGCCCGTCGCTCCTACCGATTGAATGGTCCGGTGAAGTGTTCGGATCGCGGCGACGTGGGCGCTCCGTCGCCGACGACGTCGCGAGAAGTCCACTGAACCTTATCATTTA

> [Lagerstroemia indica] Actin

ATGGCTGATGCAGAGGACATTCAGCCCCTTGTCTGTGACAATGGTACCGGAATGGTGAAGGCTGGATTTGCTGGTGATGATGCTCCTAGGGCGGTCTTTCCCAGTATTGTTGGTCGACCTAGGCACACTGGTGTTATGGTAGGTATGGGTCAGAAGGATGCATATGTTGGTGACGAAGCCCAATCCAAGCGAGGTATCCTTACCTTGAAGTATCCGATAGAACATGGTATTGTGAGCAACTGGGATGACATGGAAAAGATCTGGCATCACACTTTCTACAATGAGCTCCGTGTGGCTCCTGAAGAGCACCCTGTTCTTCTCACGGAGGCCCCTCTCAACCCCAAGGCCAACAGAGAGAAGATGACTCAGATTATGTTCGAGACATTCAATGTGCCTGCCATGTATGTTGCCATTCAGGCTGTGCTTTCCCTGTATGCAAGTGGTCGTACAACTGGTATTGTGCTGGATTCTGGTGATGGTGTGAGTCACACGGTCCCGATCTATGAGGGTTATGCCCTGCCCCACGCTATCCTCCGTCTCGACCTTGCTGGACGCGACCTGACTGATGCTCTCATGAAGATTCTCACTGAGAGAGGTTACATGTTCACCACCACTGCTGAGCGGGAAATTGTCCGTGACATGAAGGAGAAGCTTGCATATGTTGCCCTTGACTATGAGCAGGAACTCGAAACTGCTAAGAGTAGCTCATCAGTCGAGAAGAACTACGAGCTTCCTGATGGACAAGTCATAACCATCGGTGCCGAGAGGTTCCGCTGCCCTGAAGTCCTCTTCCAGCCCTCGCTGATCGGCATGGAAGCTGCTGGGATCCATGAGACTACCTACAACTCCATTATGAAGTGTGATGTGGATATCAGAAAGGATCTCTATGGTAACATTGTTCTCAGTGGTGGTTCCACCATGTTCCCTGGTATCGCCGACAGGATGAGCAAGGAGATCACTGCCCTTGCCCCAAGCAGCATGAAGATTAAGGTTGTTGCACCGCCAGAGAGAAAGTACAGTGTCTGGATTGGAGGATCGATCCTTGCGTCTCTCAGCACATTCCAGCAGATGTGGATTTCCAAGGGCGAGTACGATGAGTCGGGTCCATCAATCGTCCACAGGAAGTGCTTCTAG

> [Lagerstroemia indica] RNA polymerase II

ATGCTGCAGCACCAAATTGCTCAGTCGCCGGCGAGGCTAGGCCTCACGAGCCCCAATTCTCCGTCACTTCAGACCGCTTCTCCTCAGCCGCCGCCCAAATTCTCCTCCTCTCAACAGCCCCAGCTCCAACCGCACCCGAACCTTGCCACTGCCCCTTCTACCTCCTCGGCCCTCCTCCCCCTCCTTCCACCGCTCCCGAGGGCCCAGTCCCTCCTCCTCCAGATGGCTTCCCTCGCCACGAAGCTCTTTGAAGTCTCCCCGAACCGCTCTTTTTGGCTCACCTCATTCCGCGGAGTGTTTCCGACCTTCTTGCCATCCCAGTCGCAGACCCTGCCTGATCAGTCCTCTTCCTCCACCAAAGAGGTCCTCTCCCTTTTCACTTCTCTCCAGACCCAGCTCTTCGAGGCTGTGGCTGAGCTCCAGGAGATCCTCGACCTCCAAGATGCGAAGCATAAAGTCTCCCGAGAGGTCCGGTCAATGGACGCATCCTTGCTGTCCTTCGCCCACAAGCTTAAAGAGGCCGAGCGGGTCCTCGATGTTCTAGTGGATGATTACTCTGATTACCGCTGCCCCAAGAGGTCGAAATCGGAGGATGCCGAGGAGGAGGATGAATCTTCATTCACGACCATGGCTTCTCGGCTGAATCTCTCGGACATCATATCATATGCTCACCGGATAAGCTACACGACCTTCGCTCCTCCCGAGTTCGGGGCTGGGCAGGCACCTCTCCGTGGTGCCCTTCCTCCTGCTCCACAAGAGGAGCAAATGCGAGCCTCGCAGCTTTACAACTTTGCCGATCTGGATGTTGGGTTGCCTAAAACAGTCGAAACTAAGGAGAAGACAATCGAGCCTATCATTGAGCCAACTCTCGTCCCAGACACGAATAATCCTCTTGCAAATTTGGCAGCTATCCAGGGCCTGCTTCCTCCGAATATTACAGTTCCATCGGGTTGGAAGCCGGGGATGCCAGTGGAGCTCCCAACCAATCTGCCTGTGCCCCCACCTGGGTGGAAACCAGGGGACCCTGTCCCACTTCCTCCACTGGACTCTCTTGCTGCGCCTAGGATCGAGGAGCCGCAAATACGACCGGTCGCTCCTCAAGGCATGCACAAGGAGCCAGAGATTATACAGGTTCGGCACGTCGACCTGGAACTGCCTGATAATTTCGATGATAGCAGCGACTACAGTAGCGATGAGGGAAGCTCCGAGGAGGATGACTGA

> [Lagerstroemia indica] Cyclophilin

ATGGGCAACCCGAGGGTCTTCTTTGACATGTCGATCGGCGGCCAGCCCGCCGGTCGGATCGTGATGGAGCTCTTCGCCGACACCACCCCCCGCACGGCCGAGAACTTCCGCGCCCTCTGCACCGGCGAGAAGGGGGTCGGCCGCTCCGGCAAGCCGCTCCACTACAAGGGATCCACCTTCCACCGCGTGATCCCGGGCTTCATGTGCCAGGGCGGCGACTTCACCGCCGGGAACGGCACCGGAGGCGAGTCGATCTACGGTGCCAAGTTCGCTGACGAGAACTTCATCAGGAAGCACACCGGCCCTGGCGTCCTGTCCATGGCGAACGCCGGCCCGGGGACCAACGGATCCCAGTTCTTCATCTGCACCGCTAAGACGGAGTGGCTGGACGGGAAGCACGTGGTGTTCGGGCAGATCGTGGAGGGGATGGACGTAGTGAAGGCGGCGGAGAAGGTGGGGTCCAGCTCCGGCAGGACCTCGAAGCCGGTCGTGATCGCGGACTGCGGCCAGCTCTCTTAG

> [Lagerstroemia indica] Elongation factor-1-alpha

ATGGGTAAGGAGAAGTTTCACATTAACATTGTGGTTATTGGCCACGTCGACTCTGGTAAGTCCACCACCACTGGCCACTTGATCTACAAGCTTGGAGGTATCGACAAGCGTGTGATTGAGAGGTTTGAAAAGGAAGCTGCTGAGATGAACAAGAGATCCTTCAAGTATGCCTGGGTGCTTGACAAGCTGAAGGCTGAGCGTGAGCGTGGTATCACAATTGACATTGCCCTGTGGAAATTTGAGACAACTAAGTACTACTGCACTGTCATTGATGCCCCTGGACACCGTGACTTCATCAAGAACATGATTACTGGAACATCACAGGCTGACTGTGCTGTGCTCATCATTGACTCCACCACTGGAGGTTTTGAGGCTGGTATCTCTAAGGATGGTCAGACCCGTGAGCACGCTCTGCTTGCCTTCACCCTTGGTGTGAAGCAAATGATTTGCTGCTGCAACAAGATGGATGCCACCACTCCTAAGTACTCCAAGGCCAGGTACGATGAAATTGTGAAGGAAGTCTCCTCATACCTCAAGAAGGTGGGTTACAACCCCGATAAGATTCCATTCGTCCCAATCTCCGGGTTTGAGGGAGACAACATGATTGAGAGGTCCACCAACCTCGACTGGTACAAGGGCCCCACTCTTCTGGAGGCTCTCGACCAGATCCAGGAGCCCAAGAGGCCCTCAGACAAGCCCCTCCGTCTCCCACTTCAGGATGTCTACAAGATTGGTGGTATTGGAACCGTCCCTGTCGGTCGTGTGGAGACTGGTGTCCTCAAGCCTGGTATGGTGGTGACCTTCGGTCCGACTGGGTTGACAACTGAAGTTAAGTCAGTTGAGATGCATCACGAGGCCCTGCAGGAGGCCCTTCCCGGTGACAATGTGGGGTTCAACGTGAAGAACGTTGCTGTCAAGGATCTGAAGCGTGGGTATGTTGCATCAAACTCCAAGGATGATCCCGCCAAGGAGGCAGCTAGCTTTGTCTCCCAGGTCATCATCATGAACCATCCCGGTCAGATTGGGAGTGGATATGCCCCCGTCCTTGACTGTCACACCTCCCACATTGCCGTCAAGTTTGCTGAGCTAGTAACCAAGATCGACAGACGGTCTGGAAAGGAGCTCGAGAAAGAGCCTAAATTCCTGAAGAATGGTGATGCTGGTTTGGTTAAGATGATTCCCTCGAAGCCGATGGTGGTGGAGACCTTCTCCCAATACCCGCCTCTCGGGCGTTTTGCTGTCAGGGACATGAGGCAGACAGTTGCAGTCGGTGTCATTAAGAGTGTGGAGAAGAAGGACCCAAGTGGTGCTAAGGTCACCAAGTCTGCAGCCAAGAAGGGTGGGAAGTGA

> [Lagerstroemia indica] Eukaryotic translation initiation factor 5 A

ATGTCGGACGAGGAGCACCACTTCGAGTCGAAGGCCGATGCCGGAGCCTCCAAGACCTATCCTCAGCAGGCCGGTACCATCCGCAAGAACGGCTATCTCGTCATCAAAAACCGTCCCTGCAAGGTGGTTGAAGTTTCCACATCCAAGACTGGCAAGCACGGTCATGCTAAGTGCCACTTTGTTGCAATTGATATCTTCAGTGGCAAGAAGCTTGAAGATATTGTCCCTTCATCCCACAACTGTGATGTTCCCCATGTCAACCGCACTGACTACCAGCTGATTGATATCTCT

> [Lagerstroemia indica] Glyceraldehyde-3-phosphate dehydrogenase

ATGGGGAAGATCAAGATCGGAATCAACGGATTCGGAAGGATCGGCCGTCTGGTCGCGAGGGTCGCTCTTCAGAGAGATGACGTTGAGCTCGTTGCTGTTAACGATCCCTTTATCACCACCGACTACATGACTTATATGTTTAAGTATGACAGTGTTCACGGTCAGTGGAAGCACCACGAACTCAAAGTCAAGGACTCCAAAACTCTCCTGTTCGGCGAGAAAGCTGTCACTGTTTTTGGAATCAGGAACCCTGAGGAGATCCCATGGGCAGAGACCGGAGCTGAATACATTGTGGAGTCCACTGGTGTTTTCACCGACAAGGACAAAGCAGCTGCCCACTTGAAGGGTGGTGCCAAGAAGGTTGTCATTTCTGCCCCGAGTAAGGATGCTCCCATGTTTGTTGTGGGTGTCAATGAGAAGGAATACAAACCCGAGCTTCACATTGTGTCCAATGCTAGCTGCACAACTAATTGCCTTGCTCCACTTGCGAAAGTTATCAATGACAGGTTTGGTATTGTAGAAGGTCTCATGACTACAGTCCACTCAATCACAGCTACACAAAAAACTGTTGATGGACCGTCGATGAAGGATTGGAGAGGTGGTAGGGCTGCCTCATTCAACATCATTCCCAGCAGCACAGGAGCTGCTAAGGCTGTTGGAAAAGTATTGCCAGCACTCAATGGAAAGCTGACTGGAATGGCTTTCCGTGTCCCCACTGTTGATGTTTCAGTGGTAGACCTTACTGTGAGACTTGAGAAAGGAGCCACTTATGAGGAGGTCAAAGCTGCAATTAAAGAGGAATCTGAGGGAAAACTGAAGGGAATTCTTGGCTATACTGAGGATGATGTGGTGTCAACTGACTTTGTGGGTGATAACAGGTCGAGCATCTTCGATGCCAAGGCTGGAATTGCTTTGAACGAGAAGTTTTTGAAGATTGTGGCTTGGTATGACAATGAATGGGGCTACAGCACTCGTGTCATCGATTTGATCTGCCACATGGCCTCTGTCCATTAA

> [Lagerstroemia indica] Alpha-tubulin protein

ATGAGAGAGTGCATCTCGATCCACATCGGTCAGGCCGGTATTCAGGTCGGAAATGCCTGCTGGGAGCTCTACTGCCTTGAGCATGGGATTCAGCCTGATGGCCAGGCGCCAAGTGACAAGACTGTCAACGGAGGTGATGATGCTTTCAACACCTTCTTCAGTGAGACAGGAGCTGGGAAGCATGTTCCTCGTGCTGTTTTTGTTGACCTGGAGCCCACCGTCATAGATGAGGTGAGGACTGGAACGTATCGCCAGTTGTTCCACCCTGAGCAGCTCATCAGTGGCAAAGAAGATGCTGCCAACAACTTTGCTCGTGGCCACTACACAATTGGGAAAGAGATTGTCGATCTATGCTTGGACAGGATACGCAAGCTTGCTGACAACTGCACCGGGCTCCAGGGATTCCTTGTCTTCAACTCTGTTGGAGGTGGTACCGGCTCTGGTCTTGGCTCCCTTCTGTTGGAGCGCCTCTCTGTGGACTATGGCAAGAAATCAAAACTTAGTTTCACTGTGTACCCATCACCCCAAGTTTCCACCTCCGTCGTAGAACCATACAACAGCGTCCTTTCAACCCACTCCCTCCTTGAGCACACGGATGTTGCTGTTCTCCTTGACAATGAAGCCATCTATGACATCTGCAGACGCTCCCTTGACATCGAGCGCCCTACCTACACCAACCTCAACCGCCTTGTCTCTCAGGTGATCTCATCCCTCACTGCCTCACTGAGGTTCGATGGTGCCCTGAATGTGGATGTAACTGAGTTCCAGACCAACCTTGTGCCTTACCCAAGGATCCACTTCATGCTTTCCTCCTATGCTCCAGTCATATCAGCCGAGAAGGCCTACCATGAGCAGCTCTCCGTGGCAGAGATCACCAACAGTGCCTTCGAGCCTTCCTCCATGATGGCCAAGTGCGACCCTCGCCATGGCAAGTACATGGCCTGCTGCCTCATGTACCGTGGAGATGTAGTCCCCAAGGATGTCAATGCTGCTGTGGCCACCATAAAAACCAAGCGCACCATCCAATTTGTGGACTGGTGCCCGACTGGATTCAAATGTGGAATCAACTACCAGCCACCTGCCGTGGTTCCAGGGGGAGACCTTGCAAAGGTCCAGAGGGCCATGTGCATGATCTCCAACTCCACCAGTGTTGCAGAGGTGTTCGGTCGCATCGATCACAAGTTCGACCTCATGTATGCAAAGAGAGCGTTTGTGCACTGGTACGTGGGTGAGGGCATGGAGGAAGGAGAGTTCTCCGAGGCCCGGGAGGATCTTGCTGCACTAGAGAAGGATTATGAGGAGGTGGGAGCTGAGTCTGCTGAGGGTGAAGATGGAGATGAAGGAGATGAATATTGA

> [Lagerstroemia indica] Beta-tubulin protein

ATGAGGGAGATCCTTCACGTCCAGGGTGGGCAGTGCGGGAACCAGATCGGCTCCAAGTTCTGGGAGGTGATCTGCGACGAGCACGGCATCGACCCCACCGGCCGCTACAAGGGCAGCTCCTCCGACGGCGACATCCAGCTCGAGCGCATCAATGTCTACTACAATGAGGCCTCCGGTGGCCGCTACGTTCCCCGGGCCGTCCTCATGGACCTAGAGCCCGGCACCATGGACAGCATCCGCTCCGGCCCCTACGGCCAGATCTTCCGCCCCGACAACTTCGTCTTCGGCCAGTCCGGCGCCGGCAACAACTGGGCCAAGGGTCACTACACCGAGGGCGCCGAGCTCATCGACGCTGTCCTCGACGTCGTCCGCAAGGAGGCCGAGAACTGCGACTGCCTCCAAGGTTTCCAAGTGTGCCACTCACTTGGAGGAGGCACTGGCTCGGGTATGGGAACTCTCTTGATCTCAAAGATCAGGGAGGAGTACCCGGACAGAATGATGCTCACATTCTCTGTCTTCCCTTCCCCAAAGGTCTCTGACACAGTTGTGGAGCCATACAATGCCACCCTTTCTGTGCATCAGCTGGTCGAAAACGCTGATGAGTGCATGGTCCTTGACAATGAAGCCCTCTATGACATCTGCTTCAGAACATTGAAGCTTAGCACCCCAAGCTTTGGGGACCTGAACCACTTAATCTCTGCTACCATGAGTGGAGTGACCTGCTGCCTTCGATTCCCGGGGCAGCTGAACTCGGACCTCCGAAAACTTGCAGTCAACCTGATACCATTCCCTCGTCTCCACTTCTTCATGGTTGGGTTCGCCCCGCTGACCTCCCGCGGATCCCAGAAGTACATCTCCCTCACCGTGCCCGAGCTGACGCAGCAGATGTGGGATGCCAAGAACATGATGTGCGCCGCCGATCCCCGTCATGGCCGCTACCTTACAGCATCGGCCATGTTCCGTGGGAAGATGAGCACCAAGGAAGTGGACGAGCAGATGATCAATGTCCAGAACAAGAACTCCTCCTACTTCGTGGAGTGGATTCCTAACAATGTGAAATCAAGTGTGTGTGATATTGCACCACAGGGCCTGAAGATGGCCTCGACTTTTGTCGGAAACTCTACCTCAATTCAGGAGATGTTTAGGCGGGTGAGCGAGCAGTTTACAGCCATGTTCAGGAGGAAGGCCTTCTTGCACTGGTACACCGGGGAAGGGATGGACGAGATGGAGTTCACCGAGGCCGAGAGCAACATGAACGACCTCGTCTCGGAGTATCAGCAGTACCAGGATGCCACTGCCGATGAGGAGATCGAGTACGAGGAAGAGGAGGAACAAGAGCACATGTGA

> [Lagerstroemia speciosa] AGAMOUS 1 gene

ATGGGGAGAGGGAGAGTGGAGCTGAAGAGGATAGAGAACAAGATCAACAGGCAGGTGACCTTCGCTAAGCGGAGGAATGGGCTCCTCAAGAAAGCCTACGAGCTCTCCGTCCTCTGCGACGCCGAGGTTGCTCTCATCATCTTCTCCAATAGAGGAAAGCTGTACGAGTTCTGCAGCTCCTCCAGCATGCTCAAGACTTTGGAGAGGTACCAGAAATGCAGCTACAATGCATTGGAGCCAAATGTGTCTGCAAAAGAGGCTTCCCTGGAGCTGAGTTGCCAGCAGGAGTATCTCAAACTCAAAGCACGTTATGAAGCCCTTCAAAGAACCCAGAGGAATCTCCTCGGAGAAGAACTTGGCCCGCTTAGCAGCAAGGAGCTTGAATCCCTTGAGAGGCAGCTAGATGCATCTTTGAAGCAGATCAGATCAACAAGGACCCAATACATGCTGGATCAACTCGGAGATCTTCAAAGGAAGGAGCATATGCTCAACGAGGCAAATCAGGCTCTAAAGCAACGGTTGATGGAGGGATACCAGGCAAACATGCTGCAGCTAAATGCCGCCGCGGAGGAAGTGGGCTACGGCCGAGCGGGCGCTCCTCCACCTCCGGTCGATGGGCTCTTCCACCCTGTGGCCTGTGAGCCCACTCTTCATATCGGATATCAGCCCGATCACATGGCGGTAGTCACGGCTGCTGGGCCGAGCGTGACCAATTTCATGCCAGGATGGATGCCATGA
